# Mutual learning: exploring collaboration, knowledge and roles in the development of recovery-oriented services. A hermeneutic-phenomenological study

**DOI:** 10.1080/17482631.2021.2001898

**Published:** 2021-11-21

**Authors:** Trude Klevan, Reidun Jonassen, Alain Topor, Marit Borg

**Affiliations:** a Center for Mental Health and Substance Abuse, Faculty of Health and Social Sciences, University of South-Eastern Norway, Drammen, Norway; b Department of Psychosocial Health, University of Agder, Grimstad, Norway

**Keywords:** Recovery, mental health, substance abuse, dyadic interviews, collaborative, recovery-oriented services

## Abstract

**Purpose:**

The concept of recovery is commonly described as multifaceted and contested in the field of mental health and substance abuse. The aim of this study is to explore how understandings of recovery and recovery orientation of services are developed through daily practices and collaboration between service users and professionals.

**Methods:**

Eight pairs of participants were interviewed together, in accordance with the dyadic interview method. The dyads/pairs consisted of service users and professional helpers. A collaborative hermeneutic-phenomenological analysis was used to analyse data.

**Results:**

Data were analysed into three overarching and entangled themes, exploring how recovery-oriented collaboration and knowledge encompasses (a) recovery as relational processes. These processes are entangled with (b) recovery as situated in time and place. Furthermore, relational processes and dimensions of time and place are situated in and supported or hindered by (c) recovery orientation as part of the municipal policies, understood as the regulations, frameworks and decisions guiding mental health and substance abuse services in the municipality.

**Conclusions:**

The further development of recovery-oriented services should focus on facilitating open-ended and flexible ways of developing practices and relationships. This involves recognizing how relationships contribute to the development of knowledge and practices.

*Things take time, you know. That’s an important thing, now the municipal services are going to be recovery-oriented, recovery-based, all that stuff. There are so many fancy words they use. But what you have to realize is that what those words mean, well, that’s something you can’t finish in one day.*

This article presents a study exploring how understandings of recovery and recovery-oriented services are developed through daily practices and collaboration between service users and professionals in a Norwegian municipality. The concept of recovery is commonly described as multifaceted and contested in the field of mental health and substance abuse. Several studies emphasize that there seems to be little consensus on how recovery is to be understood and hence, how to work in recovery-oriented ways (Egeland et al., 2021; Pincus et al., 2016). However, it may be argued that a pervasive emphasis on ambiguity and diversity risks draining the concept of recovery for content. This might entail a possible “anything goes” understanding, without taking the values and roots of recovery into consideration, as suggested by the research participant in the introductory quote.

Currently, a diversity of practices and services in the field of mental health and substance abuse go by the name “recovery-oriented”. Understandings of recovery are often divided into clinically-oriented and user-oriented definitions, where the former originate from the historical context of clinical research. From this perspective, recovery is understood as primarily a clinical outcome (Slade, 2009; Tuffour, 2017). User-oriented definitions, on the contrary, perceive recovery as non-linear, personal, social, and contextual processes people engage in to overcome their difficulties. Based in different traditions and practices and having different goals, this broad division of definitions implies that in understanding recovery and recovery-orientation, there is a need to clarify from which position one is speaking. In this study, we position our understanding of recovery and recovery orientation of services within a critical mental health paradigm in line with the social justice roots of the original recovery movement (Beresford et al., 2016; Davidson, 2016; Klevan et al., 2020; Pilgrim, 2009; Rose, 2014). In a broader perspective, the original recovery movement paradigm can also be seen as part of the de-institutionalization that has characterized mental health and substance abuse services in the Western world since the 1950s (Davidson, 2021; Topor et al., 2016). The closure of psychiatric institutions and a shift towards community-based services to help people to live a life in their local community call for a more social and rights-based understanding of mental health and hence, what should be provided by services and the broader community (Davidson, 2021; Mezzina, 2014). In the context of the current study, we perceive recovery as a concept that may challenge certain parts of professional knowledge and power as well as individualistic understandings of mental distress and substance abuse. This also involves understanding recovery as unique and dynamic interactions and processes between persons and contexts, and perceiving recovery and recovery orientation of services as part of the everyday life of individuals and local communities (Doroud et al., 2015, 2018; Klevan et al., 2021; Reid et al., 2020; Sommer et al., 2021; Topor et al., 2011). Based on our positioning, we understand recovery as created and supported through shared processes, which can be developed and acted out through collaboration between service users and providers, affected by and affecting the diverse contexts that they are part of. As such, binary definitions of recovery as either clinically oriented or service user oriented are problematic and can conceal the fact that a variety of understandings, values, roles and knowledge bases inform people, collaboration, daily practices and processes of recovery.

In Norway, where this study was conducted, recovery-oriented practices and perspectives have been called for in national guidelines. Some Norwegian municipalities have mandated recovery orientation in their mental health and addiction services and have made local attempts to develop services in this direction (Kvia et al., 2021; Norwegian Directorate of Health, 2014; Pincus et al., 2016; Reed et al., 2020.). Positioning recovery within a person- and context-centred understanding and in line with the original roots of recovery, recovery-oriented services involve recognizing service recipients as experts on their situation and on what recovery and helpful help may involve, and as citizens with rights and obligations. (Hansen et al., 2020; Reid et al., 2020; Topor, 2021; Topor et al., 2011). Within the context of community services, developing helpful strategies that can support the service user in resuming a meaningful life and valued roles involves a relationship with professional helpers based on core values such as role-blurring, balancing of power, and exchange and negotiation of skills, expertise, and various types of knowledge (Klevan et al., 2020; Ness et al., 2014). Rather than being perceived as “expert/helper” and “help-receiver,” professionals and service users are considered collaborators, working together in a dynamic relationship, where they co-produce knowledge and practices about recovery and helpful help in each unique case based on a variety of expertise and experiences (Kidd et al., 2016; Klevan et al., 2020; Ness et al., 2014).

Collaboration as a core value not only concerns the service user-professional relationship but should also be a guiding value and principle in policies, service design and service distribution. This involves an awareness of the contexts in which relationships are developed, and the interplay between people, relationships and contexts (Sundet et al., 2020).

While there may be agreement that services should be recovery-oriented, service providers and users may find it difficult to understand what this involves in their daily practices (Kvia et al., 2021). Furthermore, attempts to streamline and implement recovery orientation in services may conflict with the contextual and collaborative underpinnings and values of recovery. (Karlsson & Borg, 2017; Kidd et al., 2016; Klevan et al., 2020). However, despite possible inconsistencies between ideas of recovery as implemented or developed, and having to deal with competing demands, people carry out their daily work and find ways to navigate and collaborate (Borg & Kristiansen, 2004; Chang et al., 2021; Lindvig et al., 2021). It could thus be argued that, in parallel to the idea of implementing recovery-oriented practices, practices are also, and have perhaps always been, developed through everyday collaboration between people. Understanding recovery as developed through daily practices and collaboration leads to an interest in exploring *how* this collaboration and development take place, what they involve and what they presuppose. Thus, in this study, we assume a bottom-up approach. The aim of the study is to explore how understandings of recovery and recovery orientation of services are developed through daily practices and collaboration between service users and professionals. The following research questions were developed:

1. How do users and providers of mental health and substance abuse services experience and describe collaboration and knowledge creation in recovery-oriented services?
2. What may facilitate or hinder collaboration and knowledge creation in recovery-oriented services?

## Methodology

This qualitative study has an explorative, collaborative and interpretive design with a hermeneutic-phenomenological approach. Such an approach combines important elements from phenomenology and hermeneutics, and is considered useful in studies that aim to obtain a deeper understanding of the meaning of lifeworld experiences (Hummelvoll, 2008). The phenomenological element in the current study involves an attempt to describe common components of lived experiences of collaboration and knowledge creation between service users and professionals in recovery-oriented services. The hermeneutic element is based on a recognition that experiences are part of contexts and are subject to various levels of interpretation by the participants and the researchers, before, during and after interviews. Further interpretation occurs in the analysis through the dialogue between the researchers and the texts of the interviews. A hermeneutic-phenomenological approach involves an explicit commitment to the hermeneutic nature of phenomenological research, understanding the nature of human experiences as contextually embedded and intersubjective (Boden & Eatough, 2014). While aiming to describe and explore some common components across the set of data, we also want to emphasize that in a collaborative and interpretive study of this kind, “knowledge” and “truth” are understood as interpreted and ongoing processes that are constructed and re-constructed via the interactions between the researchers, the participants and their respective contexts (Klevan et al., 2017).

The study was conducted in collaboration between the four authors. The first, third and fourth authors have a clinical and academic background in mental health. The second author is a peer researcher and social worker. In this way, our different backgrounds and experiences form part of the interpretive processes and hermeneutic movements that have contributed into all parts of this study.

## Participants

In this study, the inclusion criteria were: service users above the age of eighteen with mental health and/or substance abuse challenges who received support from community-based services in a medium-sized Norwegian municipality. Furthermore, they had to have received community mental health and/or substance abuse services for two years or longer, and to be still in contact with the services.

Eight service users, five women and three men, agreed to participate. They were recruited from a broad range of services, such as ambulatory teams, day centres and supported housing. All were currently receiving services on a regular basis. The participants also had previous experience of receiving a variety of other services. After agreeing to participate, they were asked to select the professional whom they, regardless of why, defined as being their most important helper from the service they were currently using, and invite this person to be interviewed with them in a *dyadic interview* (Klevan et al., 2020; Morgan, 2016). The invited person had to work in the community mental health and substance abuse services, but there were no specific criteria for education, profession,or the content and nature of the helping relationship. Seven of the professionals who participated were qualified in mental health and/or substance abuse, while one was a peer support worker. Three were men and five were women.

## Procedures

The aim of the study, interview guide and procedures for recruitment were developed in a “competence group” (Brekke et al., 2018; Trangsrud et al., 2021). The group consisted of people with experience as service users, mental health workers and service managers, in addition to the researchers. The group met five times during the research process to discuss and reflect on the above-mentioned issues. The group also reflected on and provided input on the preliminary analysis and findings and how these might be interpreted. The group was established to strengthen the quality of the project by facilitating a variety of forms of knowledge in parts of the research process (Brekke et al., 2018).

The eight pairs of participants were interviewed together, in accordance with the *dyadic interview method* (Klevan et al., 2020; Morgan, 2016). In this particular study, the dyads/pairs consisted of service users and professional helpers who already had a relationship. In dyadic interviews, the comments of each participant draw forth responses from the other, and data are generated through dialogue between the pair and the researcher about a topic (Morgan, 2016; Morgan et al., 2016). Thus, we considered dyadic interviews to be particularly feasible when aiming to explore the intersubjective nature of recovery-oriented collaboration and development of knowledge between service users and professionals who were involved in ongoing working relationships (Klevan et al., 2020).

To enable further in-depth exploration of issues raised in the interviews, the plan was to interview each pair twice, with the second interview building closely on the first one. Due to the COVID-19 situation, the period between the two rounds of interviews was longer than originally planned. Of the eight pairs, five were able to participate in the second interview. The data therefore consisted of 13 dyadic interviews. Data in the first round were generated using semi-structured dyadic interviews based on a thematic guide that focused on the topics of collaboration, roles and knowledge creation between the interviewed pairs, in the context of recovery-oriented services. Open-ended questions were used to elicit first-person experiences. The first round of interviews was conducted jointly by the first and second author. Interviews in the second round were shaped more like an open conversation between the researcher and the participants in the dyads. These interviews were conducted by the first author. The interviews began with the first author summarizing what had been talked about in the first interview. The pairs were then invited to share their thoughts and reflections. These interviews thus enabled elaboration on issues from the first interviews that the researchers and participants found to be important.

## Ethics

The study was carried out in accordance with the regulations of the Norwegian National Research Ethics Committee. Due to the nature of the study, the ethics committee concluded that the study did not require formal ethics approval (information removed). The Norwegian Centre for Research Data approved the study (information removed). Written informed consent was required before participation in the study, and data were anonymized during the transcription process. Asking the service user to identify and invite the person they considered their most important helper was an important part of the procedure. In relationships that can be considered asymmetrical, this procedure might reduce inequality whilst also granting people a voice and the ability to decide for themselves (Caldwell, 2014)

## Analysis

The analysis was inspired by the work of Hummelvoll (2008). A collaborative hermeneutic-phenomenological analysis does not follow a predefined set of analytical steps and procedures. Its strength and potential can be argued to lie in the iterative back and forth process between the descriptive and interpretive dimensions of the data, with the aim of developing meaning and knowledge through the dialogue between the two dimensions. The analysis can be viewed as a data-guided creative process, where different questions are posed to the text to explore its possible meanings (Hummelvoll, 2008; Klevan et al., 2017; Kvale & Brinkmann, 2015; Ödman, 2007). The analysis was conducted by the first author, with input from the other authors and the competence group. This collaboration led to multiple views and understandings of the data, enabling reflections and dwelling on either consensus or disagreement in the interpretations of the data material (Hummelvoll, 2008). However, while the analysis had a clear collaborative element, it was nonetheless guided by the first author who made the final decisions.

Following each interview in the first round, the first and second author discussed the interviews, focusing on immediate reflections and reactions. The first author then wrote down reflective notes based on impressions from the interviews and the subsequent discussion. The interviews from the first round were transcribed verbatim by the first author. The first author made a preliminary idiographic analysis of each interview, reading carefully through the transcribed material and making notes on possible themes. The preliminary analysis was then presented and discussed with the second author and the competence group, who provided input on how the findings might be interpreted. The first author’s preliminary analysis and the input and interpretations from the second author and the competence group were used to develop emerging themes from each interview which were then used as a basis for the dialogues in the second round of the interviews. These interviews were conducted by the first author. Following each second round interview, the first author took reflective notes. The second round interviews were transcribed by the first author. All interviews were then coded and arranged into preliminary themes by the first author. The second author read through the data and made notes of possible themes and interpretations. The two authors then looked at the material and the emerging themes together, developing the final themes as a collaborative process. Succeeding this, the third and fourth author were invited in, and the themes were subject to a final refinement. The entire analysis involved an iterative back and forth process between descriptive and interpretive levels (Hummelvoll, 2008).

On this basis, the analysis was not strictly sequential. Bearing this in mind, the process may be summarized through the following steps: 1) iterative reading of the material and making notes of preliminary interpretations; 2) rereading of transcripts and identifying and coding meaning units; 3) abstraction of the coded material into subthemes via a thorough revision of codes in relation to each interview and the data set as a whole; 4) a collaborative process on grouping subthemes into themes, and elaboration and refinement of the themes via an iterative back-and-forth process between the text of each interview, the text as a whole and the evolving themes; and 5) testing and refining the final themes and subthemes.

## Findings

Data were analysed into three overarching themes exploring how recovery-oriented services are developed through collaboration and knowledge creation between service users and professionals, and the conditions that are needed. The themes may also be interpreted as dynamic and reciprocal dimensions that interact with, presuppose, and depend on each other. The findings explore how recovery-oriented collaboration and knowledge encompasses ***recovery as relational processes***. These processes are entangled with ***recovery as situated in time and place***. Furthermore, relational processes and dimensions of time and place are situated in and supported or hindered by ***recovery orientation as part of the municipal policies***, understood as the regulations, formal and informal frameworks and decisions guiding mental health and substance abuse services in the municipality. In the presentation of the findings, service users are referred to as (S) and professionals as (P).

## Recovery as relational processes

This theme encompasses experiences of how recovery is enabled and supported through a wide range of relational processes between the service user and the professional. These processes were described as mutual and ongoing, resting on the unique relationship between the two, and thus emphasizing how recovery can be understood as collaborative processes. Collaborative processes appear to be based on certain rather consistent characteristics, even though recovery processes may develop over time. Recovery as collaborative and relational processes is explored through the subthemes: ***simply being human, balance between give and take***, *and **learning from each other.***

An important part of these processes was expressed through the subtheme of ***simply being human.*** This expression involved experiences of being together just as “human beings”, a state of being together that rested on mutual honesty and trust. This could be developed and maintained through sharing emotions, interests, and experiences from daily life and personal history, regardless of whether one was a service user or professional. One of the pairs described how they had known each other and collaborated over a long period of time. They had therefore gotten to know each other well, and there was a certain openness for both to share experiences from their private lives that could be used for joint reflections on how to live and deal with challenges in life:

*S:* *Well, you know, we both lost one of our parents. Almost right after each other.*

*P:* *Yes, we did.*

*S:* *And so she warned me a little (about possible reactions) … and they came.*

Simply being human together also involved a genuine desire to want the best for each other, and a mutual certainty that this was true. Doing everyday activities together was also a way of learning about the other and oneself as human beings. Seemingly small and mundane activities of everyday life were described as useful elements in getting to know and trusting each other, and in understanding the other as a person.

For example, one pair shared how, as part of their collaboration, they often did ordinary everyday activities like shopping for groceries or going for walks together. Such activities enabled what was described as just being together, as “normal human beings”, which seemed to promote well-being and helped to build a strong relationship:

S: *Yes, that’s exactly it. With “P”, especially, I felt that I could talk about normal life stuff, you know. Just get into the car, talk and have a nice time. Come back home again and laugh a bit about what we’d talked about.*

The subtheme ***balance between give and take*** was described as involving the ability of being sensitive to each other and each other’s needs and reactions. An important part of this was related to timing, in terms of sensing when to say and do what, and when to hold back, suggesting the necessity of being flexible and generous with each other. This involved a sensitivity to when it might be beneficial to make demands and have expectations, and when it was necessary to hold back and just listen.

Mutual expectations were emphasized as an important issue in the relationships, based on a sense of being honest and frank with each other and believing that one could expect something from the other. For service users, being seen as reliable and able to fulfil expectations was an important part of recovery.

*P:* *I have expectations for my clients, and I try to follow a sort of common thread, to see where we are in the development, according to your goals, where you’re heading … Have you noticed that I have expectations? Can you see that in our collaboration? S: I think that’s really important.*

For the professional, expectations of the service user provided a direction for their collaboration and could also inspire professional and personal growth. Nonetheless, there were times when expressing expectations could be contrary, and it was important just to listen and let things rest:

P: *And then listening’s important too. If “S” tells me that things are tough and difficult and goes into detail, then I shouldn’t say: “Yes, but think about the future and hope and stuff like that”. So I think listening to what people say is important. So if I talk about hope, he’ll feel like I’m not listening—he’ll say: “Yes, ok, but right now I need to talk about the difficult stuff.” Because just then it would be a mistake to say: “Let’s look ahead, let’s hope, and so on”.*

The mutual balance between give and take and the required sensitivity was also described as important when defining goals to work towards. While the participants stressed that these goals needed to be defined and “owned” by the service user, it was also emphasized that goals were relational. They were then developed rather than defined, implying that goals could be altered and adjusted over time, through collaboration and negotiations. Part of the development of goals could involve “pushing it a little”, meaning that the professional sometimes found it necessary to urge the other to move beyond the comfort zone:

P: *I pass the ball to him, and then I get it back. So it’s a kind of interaction. So I think: “Have I had the right focus now, like you’re the one in charge. I’m not the one who decides how things should be.*

This “pushing” appeared to be a two-way process, meaning that professionals might have to alter their pace and adjust it to the person they were collaborating with. This could involve holding back one’s urge for action, described by some as “working with your hands in your pocket”.

The idea of give and take meant that professionals had work and collaborate in more open-ended ways outside the regular professional-service user relationship. This was described as sometimes demanding, yet also important for both parties as it appeared to support a notion of equity and mutuality:

P: *Well, it used to be like, personal things, or stuff at home, well, I didn’t talk about that at work. But I don’t have any problem talking about my things with S, so that’s it, I feel you have to show more empathy. So it doesn’t get kind of static, like here’s the professional and here is the service user.*

In this way, recovery-oriented work could sometimes involve leaving the security of the professional role. The collaboration and mutuality involved also meant that the role of the service user could be expanded; the person was not only at the receiving end but could also provide the professional with important knowledge.

The subtheme ***learning from each other*** was connected to experiences of how recovery orientation of services could enable new understandings and new ways of perceiving knowledge and who holds the knowledge. While several participants emphasized that important aspects of being in recovery involved learning new things, getting advice, and getting new tools based on the professional’s knowledge, participants also pointed out how their collaboration rested upon an understanding that they learnt from each other. When working in ways that they understood as recovery-oriented, they described how they both depended on learning from each other, as there was no fixed knowledge or ready-made solutions in recovery. This could involve knowledge about living and dealing with mental distress or knowledge about dealing with various aspects of “life in general”:

*S:* *In a man’s world it’s not common to … meet people who can say, “I could see that I’d been doing it wrong all the time.” And that’s really interesting for me. It’s really cool. And it motivates me to want to change things too, you know. Like, it’s never too late to learn something new! P: I just think that we get so much further by not … like you’re there, and I’m here. I mean damn it, we’re both human beings. We both have a lot of experience. So over time, the two of us have really been reflecting on a lot of interesting things together!*

Many professionals described how they found recovery-oriented ways of working as rewarding, but also challenging. They could not rest assured that they had the necessary knowledge and solutions; instead, they had to be open to change and not rely on their professional assumptions. This seemingly less expertise-oriented professional role was also appreciated by service users as it could enable them to express their preferences and knowledge:

*S:* *What I really like about P is that from the very first moment that we met, he said, “I’m new here”, you know, and he’s curious about things. So he asks me about things, he doesn’t pretend to know*.

Thus, understanding recovery orientation as based on mutual learning appeared to enable a more flexible and open-ended understanding of what being a service user or a professional might involve.

## Recovery as situated in time and place

This theme explores how experiences of collaboration and developing ways of working together to promote recovery were closely interwoven with issues related to the use of and access to time and place. Whilst recovery efforts were related to a number of personal and interpersonal characteristics, the passing of time and flexibility concerning how one could relate to and spend time were crucial to developing recovery-oriented collaboration and practices. Furthermore, the possibility to work in a recovery-oriented and collaborative manner was also described as related to concrete, physical meeting places and spaces. This theme is explored through the subthemes ***the time it takes*** and ***physical space***.

***The time it takes*** was an expression frequently used by the participants, and it appeared to cover a range of issues and processes related to the importance of time as a prerequisite for recovery to take place and for collaborating in recovery-oriented ways. This involved how being in recovery and recovery-oriented collaboration could not be limited to a specific timeframe. The time needed was individual, and not an evenly flowing process.Furthermore, time was not just an issue of actual minutes and hours but was also connected to a *notion* of flexibility and having the leeway to do things at a pace and manner that felt natural. Naturally, time was linked to the relational processes described above. For instance, it often took time to build mutual trust. Setting goals or engaging in activities that could promote recovery could not normally be decided in the first meeting. Finding out about one’s interests or where one wished to be heading could be a long and tortuous process:

P: *So, if you meet a person who says something like, when I was younger, I used to swim, you know. So if you say how about joining the swimming group? Oh no, there’ll be too many people there, and he has social anxiety, like. But the thing is, maybe after a year or two, you might get the person to join the swimming group.*

The concept of time also involved being attentive to the time *before* the pairs started their collaboration, and thinking about the stories and contexts they had been part of. Spending time together was important in getting to know each other as people with stories, skills, and relationships in the past and present, and including such issues in the collaboration. Thus, personal timelines stretching back in the past could provide valuable resources for recovery work in the present and for possible futures.

One theme that emerged through the interviews was how the actual, **physical space** for collaboration and activities to take place was of great significance. As the participants in the interviews came from a variety of settings and had experience of diverse services, their ideas varied as to what was a good space for collaboration. However, certain aspects connected to space appeared to be common. These were how the place needed to provide a feeling of security and a feeling of choice and how what was considered “the best” place needed to be flexible.

The feeling of security that a place could provide was by many described as an important requirement for engaging in trusting and recovery-oriented relationships:

*S:* *It’s like, it doesn’t feel like home, but you feel comfortable here, it’s a base. Inside the four walls of this house you can … it’s comfortable here. P: I really believe that the surroundings affect how you … they can affect a meeting with someone.*

What might be perceived as a comfortable place to meet at one point in time could appear as inhibiting or uninspiring at a later date. Although meeting at home was commonly experienced as comfortable, it could also be important to leave home and get impulses from other places. This could involve going for walks, meeting in a café or on the service premises. Thus, the actual meeting places needed to be flexible and adjusted to changing needs. For example, people living in supported housing found it important to be able to alternate between the space of their private home and accessible common areas with available staff.

S: *I’m very happy about that. Here, people don’t interfere, and I can have as much free time and time alone as I want … and then I can go over there, whenever I want to. And there we can socialize a bit when I decide to be there. And there’s even a very nice patio there.*

Supportive places could be day centres run jointly by peer support workers and professionals. Such a centre could be experienced as a place to belong, where there was room and generosity for being who one was and moving at one’s own pace. In this regard, it was emphasized that service users should be invited in as partners on equal terms in the planning and design of such places.

## Recovery orientation as part of the municipal policy

This theme explores how the development of recovery-oriented collaboration and knowledge is situated in the context of the organization and guidelines of municipal mental health services. It also describes how the organization of the services is affected by how recovery is understood and vice versa. The theme is explored through the subthemes ***common ground, efficacy and streamlining*** *and **real and rhetorical recovery.***

The subtheme ***common ground*** was expressed as an understanding of the need for the concept of recovery to be related to some shared core values in the services and organization. To develop services in a recovery-oriented direction, it was seen as important that professionals, leaders, decision makers and service users shared certain ideas of what recovery was about and the purpose of recovery-oriented work. However, the participants had experienced a lack of common ground, sometimes complicating collaboration between different services and even between colleagues in the same services:

P: *Because you can call it recovery-oriented services if you say, well, we do what service users want. So you can still hide behind that word. So that word covers a huge amount of things. So we have to make the word more specific, give it a basic meaning, to get everyone in the service to work in a more recovery-oriented way.*

The concept of recovery appeared to cover a wide range of understandings and practices, often with little shared understanding of underlying values and theoretical concepts. There was a similar variety of understandings of how recovery-oriented services were to be perceived and implemented. Participants also emphasized how understandings of recovery and its core values and purposes did not seem to be established in the management and decision makers, as the participants would have preferred. One service user expressed it in this way:

*I’m thinking, do the people in the management know what recovery is? What recovery is, you know? Are they aware of what it’s really about?*

While all the pairs in the study expressed a notion that they collaborated in a recovery-oriented manner, this was not necessarily due to the ways the services were managed and organized. On the contrary, they often related their recovery efforts to finding ways of moving around rigid guidelines and routines and collaborating in ways they found useful and worthwhile, with or without the support of the management and services.

To many, recovery-oriented work was sometimes constrained by more or less articulated aims of ***efficacy and streamlining*** services. Many had found that attempts to run services more efficiently involved a lack of flexibility in how to collaborate, for instance, when many services introduced a practice of individual written decisions. These formal decisions regulated how often and for how long service users and professionals were to meet. The general rule appeared to be short-term follow-up. Many felt that this did not promote recovery, which was emphasized as subjective processes that were not linear or time limited:

S: *I think it’s important, regarding written decisions, you might be up there at the top and say we’re going to decide on x number of hours. But I think it’s important to ask the people who actually need the services, what they think. Because very often, one week you may not need any hours. But then the next week, maybe you need two hours because you have some problem you need to solve*.

However, although the use of formal written decisions was mainly found to hinder recovery-oriented collaboration, some participants also felt that having a certain framework could be useful at times. It could lead to an enhanced and more targeted way of working towards goals, emphasizing mutual expectations of the need to collaborate and focus.

The subtheme ***“real” and “rhetorical” recovery*** refers to how the participants in their reflections distinguished between recovery manifested through a whole-system recovery-oriented approach based on a shared ground of common values, and recovery as more of a word lacking true content but seemingly describing something significant. Talking of recovery and putting it on the agenda did not necessarily lead to recovery orientation of services. Words needed to be accompanied by action, in the acknowledgement that recovery orientation requires time and resources.

The “rhetorical” understanding of recovery was also described as potentially disguising an underlying focus on efficacy and as a consequence of this, pushing the responsibility for recovery over to the service user. Rather than building a shared, recovery-oriented culture, this type of “recovery” was guided by an individualized and outcome-oriented understanding of mental health issues and how to deal with these, ignoring the need to understand that recovery is subjective and might take time:

*P:* *Well, “then we can’t help you”, you know. This fragile balance is important, that things take time—That’s an important thing, now the municipal services are going to be recovery-oriented, recovery-based, all that stuff. There are so many fancy words they use. But what you have to realize is that what those words mean, well, that’s something you can’t finish in one day.*

Participants discussed and reflected on how they understood recovery as unique processes and practices, developed in partnership. This required a framework that provided the necessary time and flexibility, in addition to an open and negotiable understanding of what “help” implied:

*P:* *Thinking in terms of recovery takes more time, it’s more flexible. It’s not so rigid, it’s not like you choose between paths A, B and C. Because recovery is all about what does a good life mean to you, what does it look like, what can we do to get there. And then you suddenly have a range of choices between paths from A to Z, and that means there has to be room and time and possibilities for that. But when there’s still a system that—it’ll have to be A, B or C.*

Participants highlighted how paths to recovery could vary greatly, and services that mainly offered a fixed set of practices and possibilities often did not promote recovery. In order to be recovery-oriented “for real”, services needed to be organized according to recovery-oriented values. Just having a focus on user participation was not sufficient; recovery needed to be reflected in the actual practices of the services.

## Discussion

In this study we explore how collaboration and knowledge are experienced and developed in a Norwegian municipality that has mandated recovery orientation in its mental health and substance abuse services. The study shows how collaboration and various forms of knowledge are developed through a range of relational aspects and actions that are closely interwoven with issues related to time and place. Furthermore, the municipal policy can provide important support or barriers to processes of enhancing recovery-oriented collaboration and knowledge.

The study elaborates on how the dimensions of relational aspects, time and space, and the municipal policy interplay as non-linear and entangled processes. Several studies have aimed to define crucial elements in recovery-oriented services (Davidson, 2016; Leamy et al., 2011; Slade et al., 2014). For services to move in a recovery-oriented direction, the need for consensus on what constitutes well-functioning recovery has been suggested (Egeland et al., 2021; Jørgensen et al., 2020). However, it has also been argued that such consensus may be hard to achieve, which may be related to different paradigms, diverse knowledge bases and competing interests (Kidd et al., 2016). The findings of the current study elaborate on how understandings of what recovery “is” and hence what recovery-orientation “is” are complex, subjective and constantly in the making through collaboration and practices situated in local contexts. While it may very well be argued that the concept of recovery needs to be based on certain shared values and ideas, achieving complete consensus on what recovery and recovery-oriented practices should involve might be difficult and even contradictory to the contextual and collaborative “spirit” of recovery. In this section, we aim to further explore the complexity and entanglements in our findings through the following two topics: 1) relational and mutual creation of knowledge and 2) recovery as practices developed bottom-up.

## Relational and mutual creation of knowledge

The current study shows that in daily practices dyads consisting of service users and professionals who share a reciprocal and trusting relationship develop what could be perceived as limited recovery-oriented practices through sharing and exchanging various types of knowledge, often expressed through or reinforced by micro-affirmations (Topor, 2021; Topor et al., 2018). These micro-affirmations involve everyday gestures, actions and words that are commonly described and dismissed as “small things”, but that are often perceived by the people involved as having great importance (Klevan et al., 2017; Skatvedt, 2017). The current study suggests that recovery and understandings of what recovery-orientation involve are developed through reciprocity and mutual sharing of various types of knowledge and gestures. The study also shows how a “safe haven” of a reciprocal, trusting relationship can enable negotiations of possible micro-resistances, urging people to move outside their comfort zones and to expand their own and each other’s roles. This expansion of roles may also involve breaking with “usual practice” in services, challenging what counts as knowledge and professional practice and what being a service user involves (Borg & Topor, 2014; Topor, 2021). Thus, the interrelation between the unique relationship of the dyads and its potential to enhance recovery orientation and question existing practices cannot be considered separately from the unique relationship. Following this, attempts to standardize recovery and recovery-orientation of services may be seen as undesirable (Lindvig et al., 2021). Furthermore, the study also elaborates on how recovery and recovery-oriented relationships are interwoven with physical spaces, understood as places that support both the relationships and the recovery-oriented collaboration. This aligns with previous studies emphasizing how physical and material aspects cannot be seen as separate from recovery-oriented relationships and practices. Rather, these issues are interwoven, and thus affect and are affected by each other in centerless and non-linear processes (Doroud et al., 2018; Larsen et al., 2021). This study thus supports understandings of recovery as irreducibly relational and contextual processes (Price-Robertson et al., 2017; Sommer et al., 2021).

At an epistemological level, this suggests a need to challenge and expand individualistic and decontextualized epistemologies, moving towards a relational epistemology (Reason & Bradbury, 2008). A relational epistemology involves a recognition that knowledge is created through relationships. The dyads in the current study show how knowledge on a given topic is developed through the people’s relationships to themselves, each other and the contexts they are part of. This co-creation of knowledge is clearly expressed by the term “learning from each other”, emphasizing how recovery is not a one-way process where the professional guides the service user. The creation of knowledge about recovery and recovery-oriented practices appears to be enabled through flexibility in the understanding of roles, in how knowledge is defined and in who is the knower, resting on collaboration and sharing as fundamental principles. A collaborative understanding of how knowledge about help and recovery is created can be perceived as based on a different understanding of what type of knowledge should inform services. Rather than focusing on who is the expert and holds the most important “expert knowledge”, the focus is on how relationships and mutuality build knowledge and practices. While it cannot be denied that the people involved bring different forms of knowledge into the relationship, this also involves a recognition that knowledge is constantly constructed through collaboration and dialogue in the dyads (Klevan et al., 2020; Sullivan, 2011). According to Cohen (2018), a dyad can be understood as a centerless structure, where both sides depend on the other for their survival. Thus, it may be argued that knowledge and knowers depend on other types of knowledge and knowers in order to develop and in this way the development of shared knowledge is relational and based on mutuality.

In line with a broader understanding of how knowledge is created and of who the “knower” is, recovery and recovery orientation of services may presuppose a possible epistemological shift towards a relational and mutual epistemology. Thus, while the importance of the experiential knowledge of the service user is recognized, the research-based and theoretical knowledge of the professional also contributes to the co-creation of knowledge in the dyad. Furthermore, as shown in this and other studies, professionals also need the space to contribute their experiential knowledge; this is a sharing that appears to blur roles and power and leads to trust and mutual learning (Klevan et al., 2020; Lindvig et al., 2021). Thus, the development of recovery-oriented practices in mental health and substance abuse services may require a change in the epistemological paradigm of care, implying a need to redefine and re-evaluate traditional roles, knowledge, and collaboration (Martinelli & Ruggeri, 2020).

## Recovery as practices developed bottom-up

Understanding recovery as based on a relational and mutual epistemology and thereby challenging roles, knowledge and ways of collaborating suggests a bottom-up understanding of how knowledge and services can be developed. This suggests that practices may come first, in terms of knowledge arising from practice and practical wisdom and not the other way round. Flyvbjerg et al. (2012), drawing on Aristotle, have coined the term *phronetic social science* to describe knowledge creation based on “thinking about practice and action with a point of departure not in top-down, decontextualized theory and rules, but in ‘bottom-up’ contextual and action-oriented knowledge” (p. 286). The aim of developing bottom-up knowledge is to allow local and even tacit knowledge to emerge from practice. This type of knowledge is interwoven with context and thus, it cannot be taught a priori. An understanding of recovery as contextual and relationally developed practices that cannot be standardized and implemented in services has been elaborated on by Karlsson and Borg (2017). The authors suggest that recovery-oriented services are best developed by the people involved in their local context. While recovery-oriented practices can well be argued to be based on several common values and factors such as involvement, collaboration, empowerment and choices among treatment options, these elements will also vary with local context and culture (Chang et al., 2021; Davidson, 2016; Leamy et al., 2011).

The current study shows how such common values can also be perceived as relational issues intertwined with the unique relationship between the professional and the service user, and with their respective stories and contexts. Topor (2021) describes reciprocity, everyday life and doings as three central components in recovery-promoting relationships. These components align with the findings of the current study, suggesting that recovery and the development of recovery practices emerge *through* context-dependent, reciprocal relationships and collaboration, rather than being imposed on these. Thus, recognizing how relationships help to build knowledge and practice is crucial.

Recognizing recovery orientation as a common guiding principle for community mental health services, Howell and Voronka (2012) reflect on the paradox that while such services are often related to value-based issues, modern community services have been developed within a neo-liberal context. Services are thus partly evaluated on their short-term effectiveness and throughput. The opposition between value-based and efficacy-based principles is evident in the current study, which details how service providers and users struggle to navigate between the two. In this study, this is referred to as real recovery and rhetorical recovery. The study emphasizes how “real recovery” can be understood as value-based, relational, flexible practices developed bottom-up. “Real recovery” appears to be based on knowledge and practices that are perceived as centerless and developed *between* people collaborating in local contexts. Thus, it can be argued that it is important whether and how the framework of mental health practices facilitates collaboration and thus opportunities to create new knowledge and roles. The somewhat controversial concept of reciprocity and mutual sharing of knowledge can be understood as important prerequisites for developing recovery-oriented collaboration and services.

## Strengths and limitations

We believe that the intersubjective nature of dyadic makes the method an appropriate approach in exploring collaboration between service users and professionals. Nonetheless, like any other method, the dyadic interview has its possible limitations. A particular concern in this study was that the participants in the pairs had an ongoing relationship with defined roles as professional and service user. Thus, participants might feel that they were prevented from talking freely in the interview situation.

The current study represents a dyadic inquiry of experiences with recovery and recovery-oriented services in a specific municipality in Norway and should be considered according to this context. The purpose of a hermeneutic-phenomenological study like this one is not to de-contextualize and generalize lived experiences. The knowledge shared, developed and interpreted through the diverse processes of this study contribute to the knowledge base on how contextually situated relationships and collaboration are crucial in building knowledge and daily practices that may be perceived as recovery-oriented.

## Conclusion

The decision to develop community mental health and substance abuse services in a recovery-oriented direction does not necessarily mean that services have changed radically. In many cases, aspects that fit into a recovery-orientated framework may be part of already existing practices and understandings. However, such arguments may also be used to avoid questioning current practices, inhibit development and to carry on with “practice as usual”. The decision to focus on recovery in services may enable understandings and arguments for why and how this orientation is important and the questioning of existing practices. The current study offers valuable insight into what recovery-orientation may presuppose and involve in terms of knowledge, roles and collaboration, suggesting that thee further development of recovery-oriented services should focus on facilitating open-ended and flexible ways of developing practices and relationships. This involves recognizing how a wide range of contextually situated and interwoven experiences and knowledge that service users and professionals hold about life and “what works” helps to build relationships and hence, to the development of knowledge and practices. This recognition may involve breaking with more traditional professional and service user roles, allowing for more reciprocal relationships involving mutual learning, and influencing the development of services through various types of knowledge and bottom-up perspectives. We would argue that taking a stance at an organizational level in municipal services that recovery orientation might involve a clear break with existing organization, knowledge, roles and ways of collaborating provides an important framework for developing what in this study is labelled “real recovery”.
